# Chlorogenic Acid and Cinnamaldehyde in Breast Cancer Cells: Predictive Examination of Pharmacokinetics and Binding Thermodynamics with the Key Mediators of PI3K/Akt Signaling

**DOI:** 10.3390/biomedicines13081810

**Published:** 2025-07-24

**Authors:** Yusuff Olayiwola, Lauren Gollahon

**Affiliations:** 1 Department of Biological Sciences, Texas Tech University, 2500 Broadway, Lubbock, TX 79409, USA; yolayiwo@ttu.edu; 2 Obesity Research Institute, Texas Tech University, 2500 Broadway, Lubbock, TX 79409, USA

**Keywords:** phytochemicals, chlorogenic acid, cinnamaldehyde, breast cancer cells, Akt pathway, molecular docking, binding energy, thermostability, pharmacokinetics

## Abstract

**Background/Objective:** In the pursuit of identifying novel therapeutic agents against breast cancer, a major priority is finding agents that effectively and safely inhibit the signaling pathways sustaining cancer cells. To better focus research efforts in validating such candidates, this in silico study assessed the pharmacokinetic profiles, thermodynamics, and binding affinity of chlorogenic acid and cinnamaldehyde with the upstream mediators of the Akt pathway implicated in breast cancer cells. **Methods:** Various software and online tools were used to conduct molecular docking of the small molecules with the proteins PI3K, Akt, and PDK1, and to examine their absorption, distribution, metabolism, elimination, and toxicity (ADMET) profile. **Results:** The results show strong binding energy (all within the range of those of FDA-approved drugs) and thermostability between the compounds and the proteins. The phytochemicals were predicted to have moderate oral bioavailability and tissue distribution, and were identified as substrates of drug metabolizing enzymes, but not deactivated. **Conclusion:** Although these predictive data warrant confirmation in a biological system, they suggest that the compounds have good pharmacokinetics and are strong inhibitors of the Akt pathway, with great potential to shut down breast cancer cell invasion and migration. These data also inform more efficient experimental designs for our planned in vivo studies.

## 1. Introduction

The morbidity and mortality of breast cancer continue to rise globally despite the efforts to develop effective treatment options that will overcome the challenges con-founding the conventional therapeutic approaches [1,2,3]. In the area of therapeutic cancer research, natural products, also known as phytochemicals, have gained strong interest as promising bioactive agents that can elicit effective pharmacological capabilities against different types of malignant tumors [2,4,5,6]. Different studies have established the effectiveness and safety of various phytochemicals as antitumoral agents [2,4,5,7,8,9,10,11,12,13,14,15,16]. Breast cancer cells, regardless of the molecular subtypes, are sustained by multiple molecular signaling pathways such as PI3K/Akt, MAPK, Nrf2, Wnt/β-catenin, and NF-κB bio-signaling [7,8,9,10,11,12,13,14,15,16]. Most of the current antitumoral drugs are designed to target one or two of the components of these multiple pathways that drive the tumorigenicity of cancer cells. This results in ineffectiveness of the drug due to development of cancer cell resistance or bypassing a primary signaling pathway affected by the drug, for a secondary one [14,15,16]. Regardless of the method of resistance, it generally occurs through several different mechanisms [17]. Phytochemicals have been shown to modulate multiple pathways in cancer cells and thus “rewire” cancer metabolism, apoptosis, metastatic suppression, and destruction of cancer survivability [2,4,5,7,8,9,10,11,12,13,14,15,16]. Thus, it is critical that we better understand the pharmacology of identified, promising, bioactive agents.

Drug discovery is a complex, multifaceted process that is often hampered by such complications as drug target identification, time, cost of development, animal model limitations, low success rates, and regulatory complexities [18,19]. Nonetheless, the process is important to produce effective and safe pharmaceutical agents for prevention, treatment and/or management of pathophysiological conditions such as malignant breast tumors. Before a drug can reach the market, the drug discovery process elucidates various aspects of drug candidates such as pharmacokinetics, pharmacodynamics, and pharmacogenomics. With the current technology readily available, computer-aided drug design has become a popular approach that is now being applied in predicting drug candidates’ pharmacological profiles. This predictive, computer programming-based approach provides advantages in cutting down some of the bottlenecks that characterized drug discovery efforts [20]. Various bioinformatic resources have been developed that can provide valuable and reliable information about a potential pharmaceutical, including its absorption, distribution, metabolism, elimination, and toxicity (ADMET) profile, and thermodynamics of its interactions with identified cellular receptors [21,22].

The present predictive study employed a molecular docking approach to virtually examine interaction, and thermodynamics of the reaction, between two promising natural products, chlorogenic acid (CGA) and cinnamaldehyde (CA) [4,5], and three different proteins in the PI3K/Akt signaling pathway, phosphoinositide 3-kinase (PI3K), protein kinase B (Akt), and phosphoinositide-dependent protein kinase 1 (PDK1) [23,24,25].

**Figure 1:**
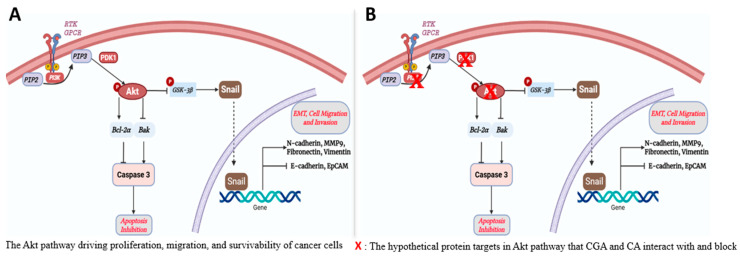
Hypothetical pathways and proteins affected by CGA and CA in PI3K/Akt signaling in breast cancer cells. (**A**) The known canonical AKT pathway. (**B**) Potential protein targets for CA/CGA intervention.

Recent in vitro investigations, in our laboratory, of the antitumor capability of CGA and CA against different breast cancer cell lines have revealed the ability of the small molecules to induce mitochondria-mediated apoptotic cell death, downregulate metastasis, inhibit invasion and migration, deplete mitochondrial membrane potential, and modulate the Warburg effect in breast cancer cells [4,5] in different molecular subtypes of breast cancer cells without significant effects to normal breast cells. The Akt pathway has been widely reported as an important signaling pathway that regulates many processes and mediators that play key roles in breast cancer (and other types of cancer) progression and aggressiveness [23,24,25]. The three important proteins upstream of the pathways are PI3K, Akt, and PDK1. The activities of these proteins result in activation of Akt by phosphorylation to form phospho-Akt (pAkt), which is then capable of modulating numerous downstream targets, both inside the cytosol and the nucleus. We hypothesized that CGA and CA would interact to inhibit these proteins, shut down the Akt pathway and its downstream signaling cascades, and ultimately bring breast cancer cell progression to a halt (Figure 1). The current molecular docking study was conducted to virtually examine the binding affinity and effectiveness of the interaction. Furthermore, we aimed to examine the pharmacokinetics ADMET profile of the natural products. This in silico assessment will provide vital information that will be important to our decision-making process as we continue our investigation of the antineoplastic potential of the two natural products. It will help us in further gathering preliminary data that are central to making informed decisions about our next xenograft mouse model of breast cancer study involving chlorogenic acid and cinnamaldehyde intervention. It will provide us with an understanding of dosing regimens based on the pharmacokinetic parameters of the phytochemicals, as well as insights into their mechanisms of action and drug–drug interaction-based safety concerns, among others. Similarly, the study will help us in using minimal numbers of mice for our in vivo investigation.

## 2. Materials and Methods

### 2.1. Retrieval and Preparation of Target Proteins and Ligands

**Figure 2:**
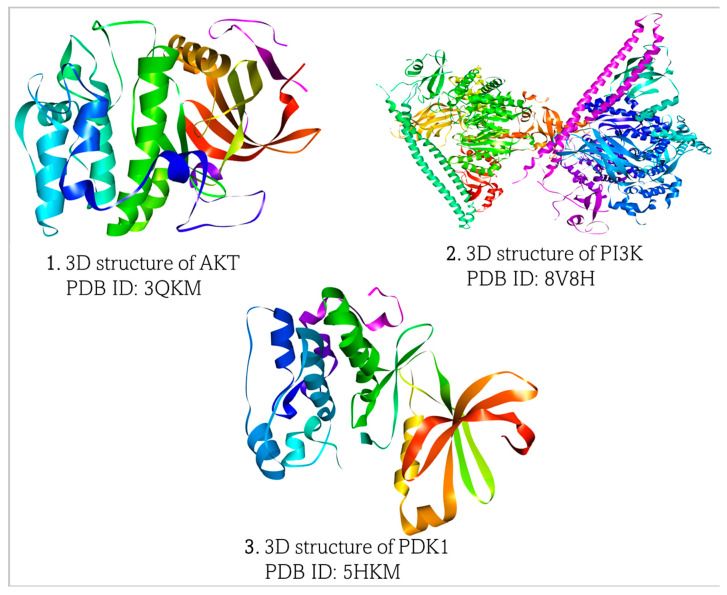
Retrieved 3D structures of Akt, PI3K, and PDK1, respectively.

Three-dimensional (3D) structures of Akt, PIP3K, and PDK1 were obtained from the Research Collaboratory for Structural Bioinformatics Protein Data Bank (https://www.rcsb.org, accessed on 7 March 2025), (Figure 2). The structures of all the proteins were generated by X-ray diffraction technique with Akt (PDB ID:3QKM), PIP3K (PDB ID:8V8H), and PDK1 (PDB ID:5HKM). The proteins were downloaded in PDB format and visualized and prepared for molecular docking in BIOVIA Discovery Studio Visualizer (https://discover.3ds.com/discovery-studio-visualizer-download, accessed on 7 March 2025).

In BIOVIA Discovery Studio, the XYZ dimensions and coordination of ligands that were co-crystallized with the downloaded protein were noted. This was important for comparison of the XYZ coordinates of the ligands under investigation that would be subsequently docked with the proteins. Next, water molecules and the co-crystal ligands were removed [26]. The retrieved proteins were prepared as .pdbqt files in Autodock Vina (https://vina.scripps.edu/, accessed on 7 March 2025). Polar hydrogen and Kollman charges were added to the protein, and Gagsteiger charges were computed [26,27].

**Figure 3:**
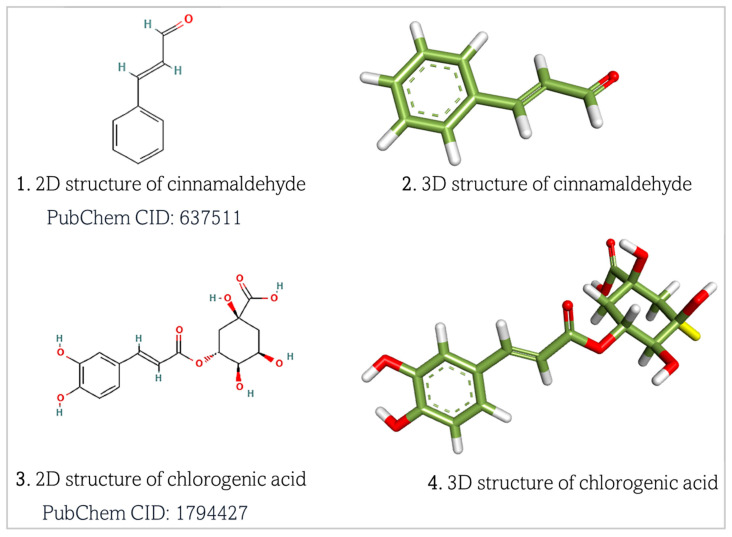
Retrieved structures of chlorogenic acid and cinnamaldehyde, respectively. Atom color meanings in the 3D molecular models: Green = Carbon (C); White = Hydrogen (H); Red = Oxygen (O). Dashed lines inside the aryl ring represent delocalized π-electron cloud. Two parallel lines represent double bonds (σ and π bonds) between atoms.

Chlorogenic acid and cinnamaldehyde were retrieved as structural data format (.sdf) files from the PubChem database (https://pubchem.ncbi.nlm.nih.gov/, accessed on 7 March 2025) (Figure 3). The retrieved 2D structures of the ligands were subsequently converted to 3D files using BIOVIA Discovery Studio Visualizer and saved as .pdb files [26,27].

### 2.2. Molecular Docking Approach

Molecular docking was carried out using two different docking software programs to ascertain the accuracy of the binding thermodynamics and pose predictions. AutoDock Vina software (version 1.2.5) (https://vina.scripps.edu/, accessed on 7 March 2025) alongside PyRx-Virtual Screening software (version 8.0) (https://pyrx.sourceforge.io/downloads/, accessed on 7 March 2025) were used to perform molecular docking for prediction of binding pose, i.e., the binding orientation and conformation, and thermodynamics of binding based on Gibb’s free energy. The docking of the ligands to the target proteins was visualized in BIOVIA Discovery Studio Visualizer.

### 2.3. Approach to Pharmacokinetic Analysis of Chlorogenic Acid and Cinnamaldehyde

The pharmacokinetic parameters, absorption, distribution, metabolism, elimination, and toxicity (ADMET) properties of the natural products were examined in silico using different ADME prediction web servers. The online resources used include ADMETlab 3.0 (https://admetlab3.scbdd.com/), Ad-metsar.v2(https://lmmd.ecust.edu.cn/admetsar2), SwissADME (http://www.swissadme.ch/), ADMET-AI (https://admet.ai.greenstonebio.com/), and bio-transformer 3.0 (https://biotransformer.ca/) (all accessed on 16 March 2025). The absorption profiles evaluated included human intestinal absorption (HIA), human oral bioavailability (HOB), and Caco-2 permeability. The distribution parameters included plasma protein binding (PPB), volume distribution, p-glycoprotein substrate/inhibitor, and blood–brain barrier penetration (BBB). The metabolic profiles included cytochrome P 450 (CYP) metabolism and inhibition and human liver microsome (HLM) stability. The excretion parameters included half-life and plasma clearance. Drug toxicokinetics was examined using Pro-tox-3.0, a virtual lab resource for the prediction of toxicities of small molecules (https://tox.charite.de/protox3/index.php?site=home, accessed on 16 March 2025).

### 2.4. Drug-Likeness Analysis

The ability of the natural compounds to act as known drug agents and elicit pharmacological impacts was examined using various web-based tools such as miDruglikeness (http://www.pkumdl.cn:8000/midruglikeness, accessed on 16 March 2025), SwissADME, Admetsar.v2, ADMET-AI, and ADMETlab 3.0. Obedience to Lipinski’s rule of five (Ro5) by the compounds was examined, as well as the compounds’ polar surface area (PSA), logarithm of partition coefficient (log P), molar refractivity, number of rotatable sigma bonds, number of heavy atoms, and number of aromatic heavy atoms.

## 3. Results

### 3.1. Predicted Molecular Docking Results

**Figure 4:**
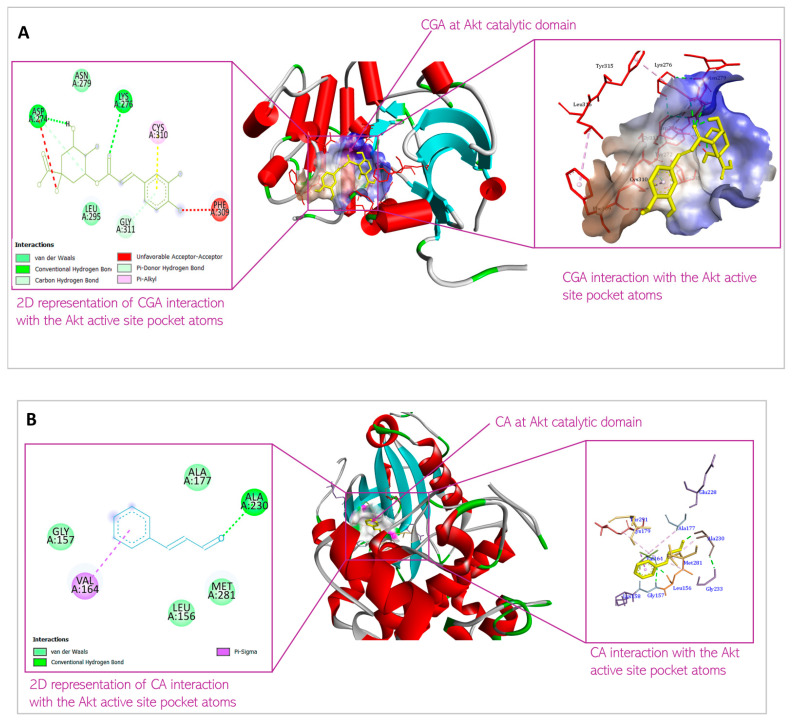
Predicted interactions of (**A**) CGA and (**B**) CA with Akt within the Akt active site.

**Figure 5:**
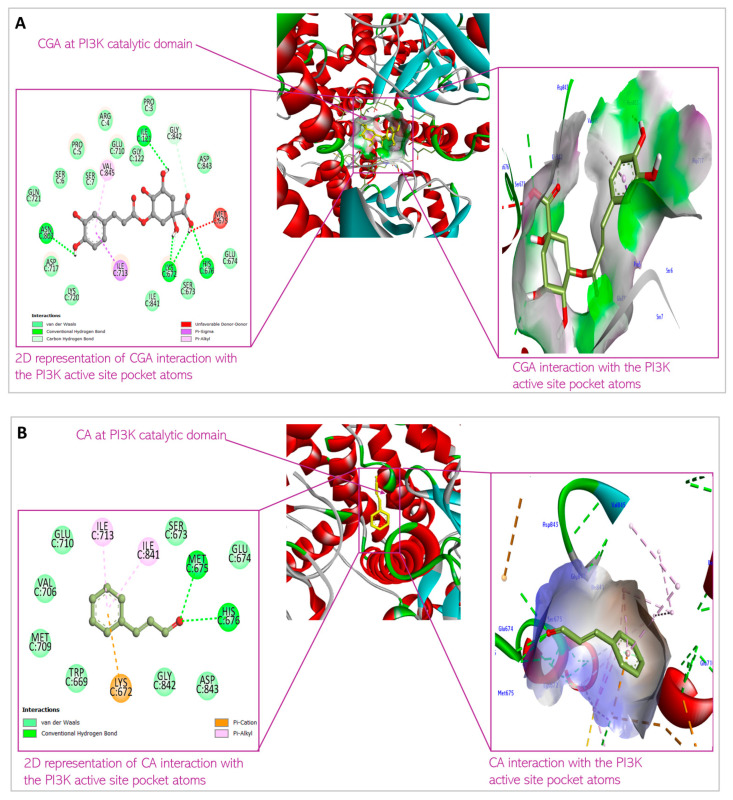
Predicted interactions of (**A**) CGA and (**B**) CA with PI3K within the PI3K active site.

**Figure 6:**
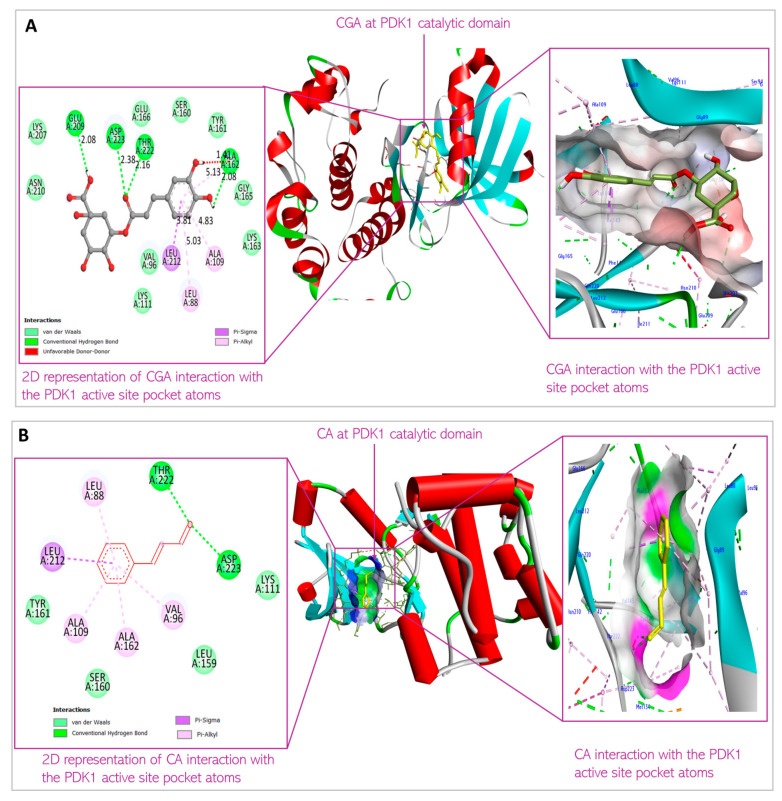
Predicted interactions of (**A**) CGA and (**B**) CA with PDK1 within the PDK1 active site.

Molecular docking was conducted to examine the binding affinity of chlorogenic acid and cinnamaldehyde to the target proteins, protein kinase B (Akt), phosphatidylinositol 3-ki-nase (PI3K), and phosphoinositide-dependent protein kinase-1 (PDK1). The docking results showed the orientations and conformations (poses) of the ligands in the binding pockets of the proteins and the amino acid residues taking part in the interaction between the ligands and the targets (Figure 4, Figure 5 and Figure 6). The binding energy revealing the thermodynamic stability of the bonding was determined (Table 1, Table 2 and Table 3). As the disruption of Akt signaling has been linked to the tumorigenicity of many types of cancer [23,24,25], the first FDA-approved anticancer agent designed to target Akt (Capivasertib) was used as a control to compare the Akt binding affinity and thermodynamics of the two phytochemical agents (Table 1). Figure 4A and Figure 4B respectively show the CGA and CA binding poses within the Akt site. The XYZ binding coordinates, binding thermodynamics, and amino acid residues with which the ligands are interacting at the protein binding sites are shown in Table 1. The binding between the individual CGA and CA and PI3K are shown in Figure 5A and Figure 5B, respectively. Table 2 indicates the binding XYZ coordinates, binding energy, and amino acid residues at the protein binding sites interacting with the ligands. Similarly, the respective CGA and CA binding poses within the PDK1 binding site are indicated in Figure 6A,B, while the binding XYZ coordinates, binding energy, and amino acids with which the ligands are interacting, are shown in Table 3.

### 3.2. Predicted Pharmacokinetic Results for Chlorogenic Acid and Cinnamaldehyde

Central to drug discovery efforts is understanding the drug pharmacokinetics, as it addresses how the body processes xenobiotics. Drug pharmacokinetics include absorption, distribution, metabolism, and excretion of the drug, as well as the toxicokinetic analysis [21,22]. Absorption is the measure of drug reaching systemic circulation through the route of administration, which could be oral, intramuscular, intravenous, subcutaneous, or transdermal. Drug absorption determines the drug bioavailability indicative of the proportion of the parent drug reaching system circulation in its active form. Drug distribution measures both the drug plasma concentration, the amount of drug that remains bound to the plasma proteins such as albumin, which could limit the drug reaching the target site, and the drug volume distribution, or the amount of the drug reaching the extravascular tissues (including the drug target receptors). Metabolism refers to the enzymatic reactions the drugs undergo to bio-transform them into more hydrophilic forms to aid their removal from the body [21]. Drugs usually undergo phase I and phase II reactions. Phase I reactions are redox and hydrolysis reactions catalyzed by the cytochrome P 450 (CYPs) enzyme system, flavin-containing monooxygenases, and various other oxidases and reductases to render the drug hydrophilic. Phase II reactions are conjugation reactions where molecular moieties, functional groups, are introduced to the drug agents to further increase their polarity, in reactions catalyzed by UDP-glucuronosyltransferases (UGTs), sulfotransferases (SULTs), glutathione S-transferases (GSTs), N-acetyltransferases (NATs), and methyltransferases, for example. Drug metabolism could result in bioactivation of the drug to become more potent, or even more toxic. Drug deactivation could also occur due to actions of drug metabolizing enzymes [21,22]. Some drugs, however, remain intact in this process prior to elimination from the body. Excretion describes the removal of the drug from the body, usually via the renal route but also through other routes such as skin and lungs. Drug clearance rate is affected by drug half-life, which is impacted by the drug binding to the plasma protein [21,22].

**Figure 7:**
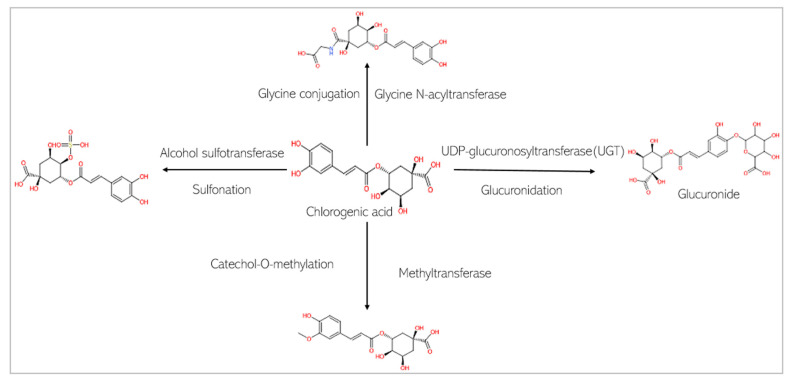
Phase II metabolism of chlorogenic acid by conjugation enzymes.

**Figure 8:**
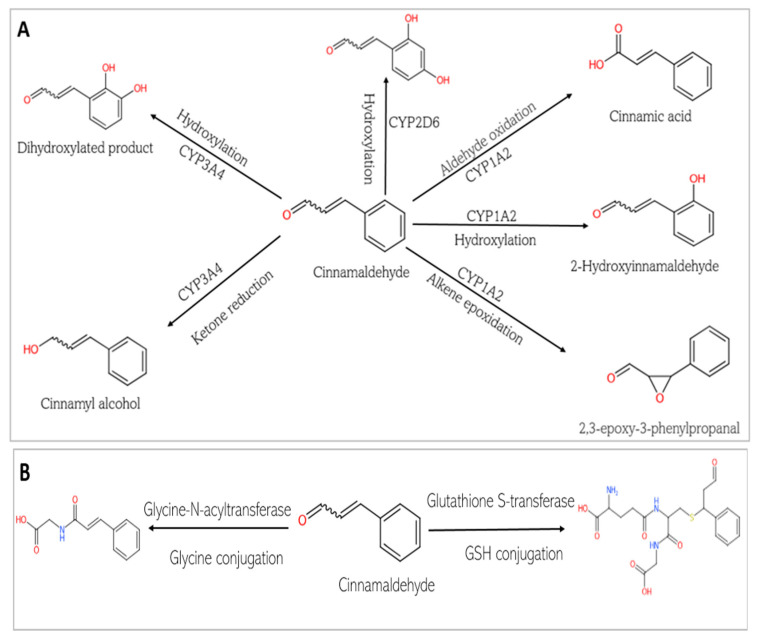

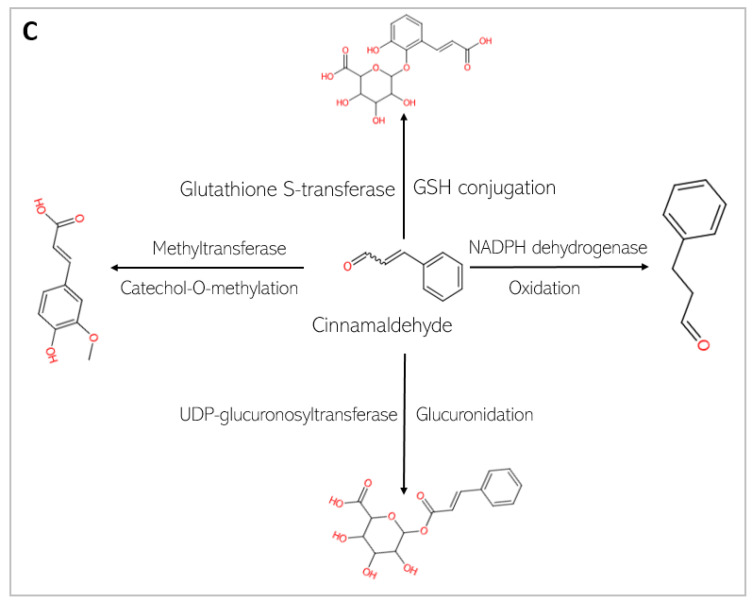
Phase I and Phase II metabolism of cinnamaldehyde by conjugation enzymes. (**A**) Phase I metabolism of cinnamaldehyde by cytochrome P 450. (**B**) Phase II metabolism of cinnamaldehyde by conjugation enzymes. (**C**) Phase II metabolism of cinnamaldehyde by human gut microbiota enzymes.

The in silico assessment of the two phytochemicals revealed their pharmacokinetic profiles, which are influenced by their physicochemical characteristics. Table 4 and Table 5 show the absorption and distribution profiles of CGA and CA. Figure 7 and Figure 8A–C show the predicted metabolic fates of both CGA and CA under the activities of the various xenobiotic metabolizing enzymes (XMEs). It should be noted that chlorogenic acid is predicted not to undergo phase I metabolism, as discussed below.

Table 6 and Table 7 show the predicted CGA and CA pharmacokinetic parameters of elimination and toxicological profiles. The drug-likeness of the two compounds, which depends on their chemical characteristics, is listed in Table 8 below.

## 4. Discussion

### 4.1. Molecular Docking

Despite improvements in breast cancer detection and more targeted treatment options, breast cancer has become a global challenge [2,3]. To develop effective therapeutic modalities against breast cancer, phytochemicals are being considered due to their re-ported efficacy and safety [4,5,28]. Whether considered as a first-line therapy or an adjuvant therapy, it is essential to understand the pharmacokinetics and pharmacodynamics of a potential pharmacological agent [29]. Similarly, identifying the interaction between a drug candidate and target proteins that play key roles in pathophysiological processes is crucial in the study of drug pharmacodynamics [30]. In breast cancer cells, the roles of the PI3K/Akt pathway in their progression and survival have been well documented, and members of the signaling cascade have been identified for anti-cancer therapies [5,23,31,32,33,34,35]. Several phytochemical agents are being tested against the pathway using different in silico, in vitro, and in vivo experimental systems [2,5,12,36]. Due to challenges encountered in drug discovery and the development process, such as extended length of time required, complexity of the process, uncertainty of the outcome, cost of drug discovery, and associated governmental regulations, predictive in silico systems have become an initial and intrinsic aspect of the contemporary drug discovery effort, to guide and accelerate the process. As a result of recent advances in computing power and technological capabilities, an in silico approach can provide rapid screening and prediction of potential drug candidates’ effectiveness and safety. This approach should hypothetically streamline the amount of time and money spent on drug discovery [20].

The current in silico study was aimed at predictive examination of two natural products, chlorogenic acid and cinnamaldehyde, for their pharmacokinetic profiles and to conduct molecular docking with Akt, PI3K, and PDK1 proteins involved in the PI3K/Akt signaling pathway. This pathway is an important target; studies have shown that inhibition of the AKT pathway can directly impact the mTOR pathway, as AKT is an upstream regulator of mTOR signaling [25,33]. Specifically, AKT phosphorylates and activates the TSC complex, which normally inhibits mTOR [25,33]. Thus, when AKT is inhibited, mTOR activation can be reduced, impacting cell growth, proliferation, and metabolism. Metformin downregulates mTORC1 via the AMPK pathway, while paradoxically upregulating the activities of Akt. It is also true that metformin, which is now reported to possess anticancer properties, can downregulate Akt activation [37].

It is important to stress that this dual role of metformin seems context-dependent. Metformin was reported to inhibit bladder cancer cell migration and growth and promote apoptosis via the inhibition of both AKT and mTOR proteins [38]. Amin et al. (2019) reported that the drug is also able to suppress mTORC1 signaling to reverse tumor growth [39]. Additionally, its impact in suppressing the expression of the protein in colorectal cancer has also been reported by Thent et al. [40]. 

In contrast, its ability to stabilize and upregulate the activity of Akt was reported in cases of postoperative cognitive dysfunction. The drug was also reported to promote osteogenic differentiation and improve ROS-driven oxidative damage of osteoblasts via activation of Akt bio-signaling [41]). Furthermore, Hu et al. (2016) reported that in an insulin-resistant rat model of NASH and cirrhosis, metformin improves the hepatic condition via IRS2/PI3K/Akt signaling, with increased phosphorylation and activation of Akt [42]. Metformin and other AMPK activators like AICAR have been reported to activate AKT through AMPK activation in certain instances, as indicated in previous investigations [42,43].

Thus, the therapeutic impact of this drug on Akt activity is arguably tissue microenvironment-dependent, with Akt downregulation observed in a tumor microenvironment. Thus, in a tumor microenvironment, with Akt downregulation, the mTORC pathway would also be suppressed. Therefore, as Akt activation is suppressed by the natural products under investigation, it is likely the mTORC pathway is also suppressed. While our previous in vitro study has indicated that Akt is suppressed, future studies will include the status of mTORC as well. 

Molecular docking is important to understand the binding affinity of drug candidates to target proteins to help predict the binding energy and thus, the thermodynamics of interaction between the drug agent and target protein. It equally reveals the potential orientation and conformation of the ligands under investigation in the binding site of the target proteins or receptors [21]. In this present study, CGA and CA were individually docked with Akt, PI3K, and PDK1 proteins. Figure 4, Figure 5 and Figure 6 and Table 1, Table 2 and Table 3 show the interaction of the small molecules with the amino acid residues at the target protein binding sites and the kind of transient non-covalent bonding holding the molecular species together.

**Figure 9:**
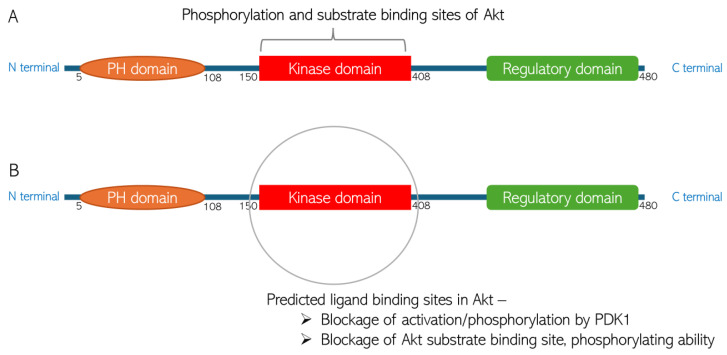
Predicted chlorogenic acid and cinnamaldehyde binding interactions with Akt. (**A**) The domain structure of Akt indicating the phosphorylation and substrate binding site in the protein kinase domain. (**B**) Representation of predicted ligand binding site in the kinase domain of Akt structure.

CGA interaction with Akt is mediated by Asp 274, Lys 276, Asn 279, Leu 295, Phe 309, Cys 310, and Gly 311. In contrast, CA interaction with Akt is mediated by Leu 156, Gly 157, Val 164, Ala 177, Ala 230, and Met 28. While all these amino acids are present in the kinase domain of Akt (Figure 9), they do not overlap. The Akt kinase domain contains amino acid residues phosphorylated by PDK1 and serves as a binding site for substrates that Akt phosphorylates. The binding of the small molecules to this domain of the protein suggests that the Akt activation (by phosphorylation) by PDK1 would therefore be inhibited, and Akt downstream targets would consequently not be modulated (activated or deactivated, based on function) by the inactive Akt. Thus, the small molecules demonstrate the ability to shut down Akt signaling via this mechanism. The binding of CGA and CA to the kinase domain of Akt is mediated by non-covalent bonding such as van der Waals forces, conventional H-bonds, π-σ bonds, alkyl-π bonds, C-H bonds, and π-donor H bonds. Such weak non-covalent bonds allow the compounds to bind to Akt without necessarily forming permanent, strong chemical bonds, and they are important for the specificity of the ligand binding to the protein.

The binding energies of the bonds are −8.2 kcal/mol and −5.4 kcal/mol for CGA and CA, respectively. The binding energy of the interactions is suggestive of the thermodynamic stability of the bonds under physiological temperature. The binding energy for the FDA-approved drugs usually ranges between −5.63 kcal/mol and −6.85 kcal/mol [22]. This is indicative of the thermostability of the bonding between the small molecules and the Akt protein. The XYZ coordinates of the small molecules are similar to the XYZ coordinates of the spirocyclic sulfonamide inhibitor co-crystallized with the Akt protein used for the molecular docking, suggesting appropriate orientations of the compounds within the Akt 3D structure. The slight difference in the XYZ coordinates is, however, expected, as the compounds have different 3D structures. Interestingly, as can be seen in Table 1, the coordinates and the binding energies of the two compounds are not far off from those of Capivasertib, the first FDA-approved anti-breast cancer drug designed to inhibit Akt [44]. As shown in Figure 9, the site in the Akt structure to which the ligands bind is the protein kinase domain. This domain consists of the amino acid residue that is phosphorylated by Akt activator (PDK1); thus, this predicted binding means Akt activation will be inhibited. Similarly, the kinase domain consists of Akt substrate binding sites. Thus, the ligand binding would block the Akt’s ability to bind and modulate its substrates.

**Figure 10:**
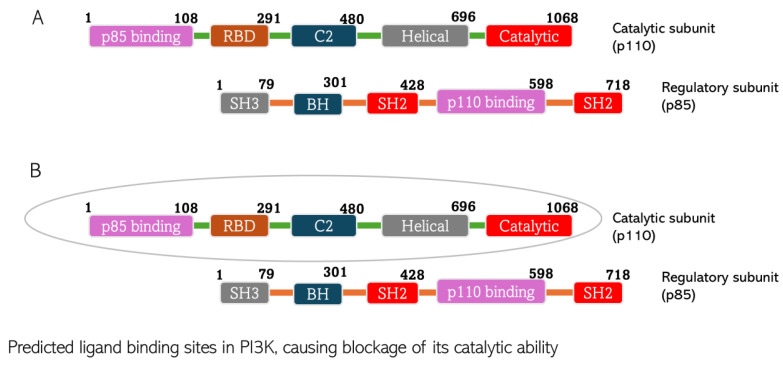
Predicted chlorogenic acid and cinnamaldehyde binding interactions with PI3K. (**A**) The domain structure of PI3K indicating both catalytic and regulatory subunits of the protein. (**B**) The catalytic subunit of PI3K predicted to constitute the ligand binding site of the protein.

CGA interacts with PI3K with the non-covalent bonding mediated by Ser7, Ile121, Lys672, Ser673, Met 675, His676, Ile713, Asn803, and Val845. CA’s interaction with PI3K is mediated by Trp669, Lys672, Glu674, His676, Val706, Met709, Ile 713, Ile841, Gly842, and Asp 843. Looking at the hetero-dimeric structure of PI3K (Figure 10), it becomes evident that the two small molecules bind to the catalytic subunit of the proteins. Interestingly, the ligands bind to multiple domains of the subunit, including N-terminal adaptor binding domain (ABD), Ras-binding domain (RBD), and kinase domain of the catalytic subunit (p110) of the protein. The predicted binding by the small molecules would produce inhibitory effects on the protein and therefore block the catalytic activity of PI3K to convert phosphatidylinositol 4,5-bisphosphate (PIP2) to phosphatidylinositol 3,4,5-trisphosphate (PIP3). The PI3K activity of phosphorylating PIP2 to form PIP3 is an important step in initiating the PI3K/Akt pathway. Thus, the inhibition of the enzyme would shut down the Akt signaling cascade. The binding is thermodynamically favorable considering the Gibb’s free energies of the reactions: −8.5 kcal/mol and −6.2 kcal/mol for CGA and CA, respectively. When comparing the XYZ coordinates of the small molecules in the binding pocket of the protein to the pyridopyrimidinones inhibitor co-crystallized with the protein used for docking, we observed similar poses between all the ligands, which are indicative of good binding affinity of CGA and CA to the protein and thus, the ability to block the catalytic impact of PI3K in Akt signaling.

**Figure 11:**
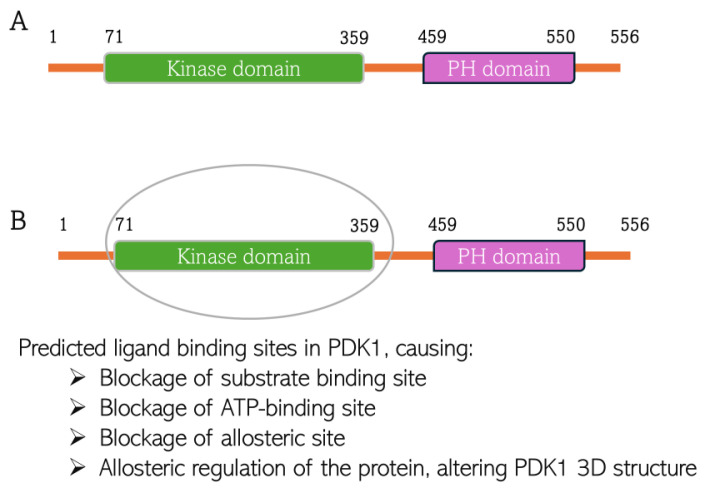
Predicted chlorogenic acid and cinnamaldehyde binding interactions with PDK1. (**A**) The domain structure of PDK1 indicating both kinase domain and pleckstrin homology (PH) domain. (**B**) Indication of predicted ligand binding site in the kinase domain of PDK1 structure.

CGA interacts with Leu88, Val96, Ala109, Lys111, Ser160, Ala 162, Glu209, Leu212, Thr222, and Asp223 at the binding site of PDK1. CA interacts with Leu88, Val96, Ala109, Lys111, Leu159, Ser160, Ala162, Leu212, and Thr222 at the PDK1 binding site (Figure 11). This is the first time overlap has been observed between the phytochemicals interacting with the AKT protein. These amino acid residues are parts of the amino acids residing at the N-terminal kinase domain of the protein. This domain, also known as the catalytic domain, consists of the substrate-binding site, ATP-binding site, and allosteric site. The ligands binding to this domain are predicted to have cellular consequences, as they would block the kinase activity of PDK1 and can induce conformational change to the 3D structure of the protein when bound to the allosteric site of the protein. The binding is predicted to be thermodynamically stable, considering the binding energy of the interactions (−8.2 kcal/mol and −5.8 kcal/mol, respectively, for CGA and CA) indicates the reactions would proceed spontaneously, exothermically. The value of the XYZ coordinates is also not far from that of the XYZ coordinates of the 7-azaindoles inhibitor co-crystallized with the protein used for the docking. This suggests the compounds are well oriented within the binding milieu of the protein.

### 4.2. Pharmacokinetics of Chlorogenic Acid and Cinnamaldehyde

Pharmacokinetics, the movement of xenobiotic agents into, through, and out of the body, is characterized by drug absorption through the route of administration into systemic circulation, distribution around the body and into extravascular tissues, metabolism by phase I and phase II drug-metabolizing enzymes, and elimination from the body, as well as the toxicological impacts of the drug agents [22,23]. As drugs are often preferred to be administered orally, drug pharmacokinetics is aimed at presenting detailed logical ADMET of the drug from the point of administration in the mouth [22,23].

Absorption is largely dependent on the physicochemical properties of the drug candidates and is defined as the ability of the drug to cross cell membranes, either via para-cellular or transcellular route, into the blood stream [23,38]. Firstly, CGA is a weak acid (pKa 3.5 to 8.6) and therefore, per pH partition theory driven by the Henderson Hasselbalch equation [45], would predictably remain unionized in the stomach of acidic pH (pH 1.2–2.0). This could enhance its cell permeability in the acid milieu of the stomach to some degree via diffusion, especially considering its small molecular size, and its delivery to the systemic venous system [22,23,45]. CGA would exist in ionized form in alkaline regions of the gastrointestinal tract (GIT) such as the large intestine. However, it would also remain in a unionized, absorbable form in the small intestine (near-neutral pH) and experience major absorption in the enterocytes, subsequently being delivered to the hepatic portal veins, from where it can enter systemic circulation [45]. CA, on the other hand, is more of a neutral compound and predictably would only be absorbed at the approximate-neutral pH of the small intestine before entry into the hepatic portal veins [22,23,45]. While CA’s transport across the cell membrane may be possible by passive diffusion considering its hydrophobicity and small molecular size, the intracellular uptake of CGA may be mediated and facilitated by a transport mechanism involving membrane transport proteins, considering its lipophobicity as dictated by its polarity and the functional groups present in its chemical structure, as discussed below.

With molecular weights of less than <500 g/mol, upon oral administration, CGA and CA may be able to transverse the plasma membrane in the GIT by passive diffusion and/or facilitated diffusion, where they would then enter the hepatic portal vein into the hepatic tissue. At this point, they can be transported via the inferior vena cava to the heart for distribution through systemic circulation. That the compounds have less than ten (10) rotatable bonds, as seen in Table 8, per Veber’s rule, confers conformational flexibility on them to permeate the apical side of the simple columnar epithelial cells, the enterocytes of the GIT mucosa, exit the basolateral side of the enterocytes, and cross into the network of blood vessels underneath the lamina propria, entering the hepatic portal veins by predictably diffusing across the endothelial cells of the vasculature [46].

While Caco-2 permeability and octanol-water partition coefficient values suggest low lipophilicity and membrane permeability of the small molecules, the results show that CA and CGA are not substrates of P-glycoprotein efflux pumps (types of ATB-binding cassette (ABC) transport proteins responsible for multidrug resistance in cancer cells) [47,48]. This indicates that simple diffusion may be inefficient for absorption of the small molecules into the cells, suggesting that specific membrane transport proteins may be required for their cellular uptake. We hypothesize that solute carrier (SLC) proteins, specifically organic anion transporting polypeptides (OATPs) such as OATP1B1, 1B3, and 2B1 isoforms, may be responsible for the cellular intake of the compounds, especially the polar chlorogenic acid. This is because these proteins are known to be ubiquitously expressed in tissues, such as hepatic, small intestinal, and mammary tissues, and are found to be upregulated in breast cancer cells with promiscuity in transportation of myriads of xenobiotics including phenols [47,48,49,50]. Further investigation to confirm this hypothesis is necessary. As of the time of this manuscript preparation, we have examined the intracellular uptake rate of CGA and CA in a breast cancer cell line, MDA-MB-231 cells, and normal mammary cell line, MCF10A cells. What is next for us, therefore, is to conduct investigation to verify the roles of the OATP isoforms, and other potential SLCs, using in vitro systems in our laboratory to elucidate the mechanism of intracellular uptake of the compounds.

Drug distribution is a key factor in determining if—and how much of—a drug will reach extravascular tissues to elicit its pharmacological effects. It is dependent on drug plasma protein binding, volume distribution, tissue mass, tissue blood perfusion, and other factors [51]. The results of our predictive study show that CGA has moderate plasma protein binding, while CA is predicted to be highly bound to the plasma proteins. These differences indicate the expected degree to which they can be distributed to extravascular tissues from the systemic circulation. The phytochemical agents are also predicted to have low volume distribution values (Table 5), which suggests the drug will most likely remain in the blood plasma for a longer period, and a lower amount will be available to reach target tissues. In the light of these predicted values, an increase in drug dosage should be considered to achieve a higher tissue concentration of the drug candidates to elicit their therapeutic effects. The breast is composed of highly perfused tissues [52,53], with networks of vascular systems mediating blood flow in and out of the tissues. Thus, drug agents from systemic circulation could be adequately delivered to the tissues to affect the tumor microenvironment.

This study predicts that the compounds are substrates of drug metabolizing enzymes (Figure 7 and Figure 8). CA is predicted to be metabolized by three isoforms of cytochrome P 450s (CYPs): CYP1A2, CYP3A4, and CYP2D6. While phase I reactions can either bioactivate or deactivate a xenobiotic, CA, is predicted to produce metabolites that possess bioactivity similar to that of the original parent compound. These include 2-hydroxycinnamaldehyde, cinnamyl alcohol, and cinnamic acid (Figure 8A). However, an unwanted toxic metabolite, 2,3-epoxy-3-phenylpropanal, is also produced (Figure 8A). Interestingly, the highly reactive cellular epoxides can be hydrolyzed to the less reactive dihydrodiol form by epoxide hydrolases (commonly present in cells), thus rendered inactive and polar, at which point they are removed from the cells [54]. CA, similarly, is a substrate of phase II conjugation enzymes (shown in Figure 8B). The enzymes, Glycine-N-acyltransferase and glutathione S-transferase (GST), bind molecular moieties (glycine and glutathione) to their substrates to increase the water solubility of the parent compound, facilitating removal from the body. Furthermore, CA is predicted to be a substrate of human gut microbiota metabolic enzymes including NADPH dehydrogenase, methyltransferase, UDP-glucuronosyltransferase (UGT), and GST (Figure 8C). CGA, on the other hand, is not predicted be to be a substrate of any of the CYP isoforms and is therefore not expected to undergo a phase I oxidoreduction reaction. However, it is predicted to be acted upon by various phase II enzymes, including alcohol sulfotransferase, glycine N-acyltransferase, methyltransferase, and UGT (Figure 7).

Since the compounds become significantly polar upon being metabolized by enzymes of phase II reactions, then it is expected that they can be eliminated via renal routes [55]. Table 6 shows the volume of blood that would be cleared of the drug agents per unit time, referred to as plasma clearance rate, which is directly related to the rate and quantity of drug removed from the body (called drug elimination). CGA and CA, respectively, have high and moderate clearance rates from systemic circulation. This suggests time in circulation, correlating to how long their biological effects would be exerted, including potential toxicity. This would also inform the dosage to be administered to achieve a sustained effect from the phytochemicals or to lessen their potential toxicity, if needed [56]. The drug half-life values for both CGA and CA show that both compounds would be removed from the body within a short period of time [57]. Having a short half-life is beneficial for drug agents that are known to be cytotoxic. For plant-based bioactive agents such as CGA and CA that are known to elicit little to no off-target impacts, a short half-life will necessitate increasing concentration to achieve sustained therapeutic benefits of the bioactive agents.

Drug-likeness is a property of potential drugs usually considered during the drug discovery process and includes physicochemical properties that are known to impact drug behaviors in an in vivo system, such as drug solubility, membrane permeability, metabolic stability, reaction thermodynamics, transporter effects, etc. [58]. The interactions between drug agents and target proteins are usually marked by non-covalent bonds. Such weak, non-covalent interactions include hydrogen bonds. Thus, the numbers of H-bond donors and acceptors, as well as the number of electronegative elements present in a drug, are predictive of drug polarity and the number and strength of such H-bonds and other weak bonds that could be formed [58]. These, taken together, can affect factors such as the oral bioavailability and thermostability of interaction between a drug and target proteins, as well as the membrane permeability of the drug [58]. According to Lipinski’s rule [59], CGA has an appropriate number of H-bond donors and acceptors, unlike CA, as can be seen in Table 8. This may explain the higher water solubility of CGA and the higher Gibbs free energy inherently available in CGA to form bonds with the amino acid residues at the ligand binding sites of Akt, PI3K, and PDK1, to which the compound was docked, when compared with CA. Additionally, the good pose of CGA within the binding sites of the three proteins it docked with could be explained by its ability to form multiple hydrogen bonds, among other factors. While CA may not be as polar as CGA for the reason enumerated above, the compound was able to form H-bonds as well as other types of non-covalent bonds with the target proteins to which it was docked, conferring stability to the bonds between CA and the proteins, as reflected in the binding energy of the interaction between them in Table 1, Table 2 and Table 3. Topological polar surface area (TPSA) is a drug-likeness parameter that can impact the polarity and the membrane permeability of drug agents. It is described as the amount of the drug surface area covered by polar atoms [60]. As Table 8 reveals, the CGA surface is covered by a greater number of polar atoms, compared to CA. This could explain the observed polarity difference seen in the two compounds and is thus suggestive of their predicted membrane permeability. CA would be more membrane-permeable than CGA under the same pH conditions, based on this TPSA value [60].

The number of rotatable sigma bonds is a chemical property of a compound that provides information about the conformational flexibility of the compound and its ability to penetrate the membrane. Rotatable bonds are non-terminal, non-hydrogen bonded, and non-ring flexible sigma bonds that can cause molecular rotation on the axis of the atoms to which they are attached [61]. According to Veber’s rule [61] for a good drug candidate, the number of rotatable bonds is expected to be less than 10. Higher numbers can make the compound highly flexible to the point that membrane permeability becomes difficult, and posing within the target receptor becomes inappropriate. The result of this predictive examination showed both CGA and CA to have acceptable numbers of rotatable bonds (Table 8). The numbers of heavy atoms and heavy aromatic atoms are predictors of the ability of a compound to form appropriate bonds with the target protein. Appropriate numbers (Table 8) suggest good binding affinity and target selectivity of the ligands. Table 8 shows that both CGA and CA have appropriate numbers of heavy atoms, which explains the ligand binding efficiency and their ability to interact with target proteins with less binding energy [62]. The two compounds have slightly higher numbers of heavy aromatic atoms that may affect the amount of energy required to bind strongly and specifically to the targets. However, these values are only slightly above the optimal value range associated with better drug-like properties and increased developability of a drug candidate, as shown in Table 8.

The two compounds examined in this predictive examination have revealed anti-cancer characteristics in our pilot in vitro breast cancer studies [4,5]. This present in silico study provides predictive information about the pharmacokinetics of both CGA and CA, and thermodynamics of the reaction between the duo, as well as interactions with target proteins that are known to be crucial to breast cancer progression, invasion, metastasis, and aggressiveness.

We previously reported that in triple negative MDA-MB-231 breast cancer cells, and the luminal breast cancer subtype cell line MCF7, the CGA and CA combination significantly affected proliferation and caused apoptotic cell death, inhibited cell migration, and shut down the invasive ability of the breast cancer cells, inhibited AKT phosphorylation, and altered the expression levels of EMT transition markers. Therefore, the next line of action is using the data obtained from this in silico study to guide our planning for in vivo examination of the antineoplastic capacity of these phytochemicals.

## 5. Conclusions

This in silico examination revealed the ability of chlorogenic acid and cinnamaldehyde to effectively bind to PI3K, Akt, and PDK1. The binding is thermodynamically favorable, and the small molecules block the crucial domains in the protein structure and are thereby predicted to shut down the PI3K/Akt pathway. Similarly, the pharmacokinetic examination showed that the compounds have a moderate ADMET profile, as determined by their physicochemical properties. While previous studies have shown the antitumoral capacity of the small molecules [2,4,5,30], the predicted pharmacokinetics warrant confirmation under in vitro systems before moving on to in vivo investigations of the pharmacological capacity of the small molecules. As of the time of writing this manuscript, we were engaged in generating data, under in vitro settings, about the impact of gastrointestinal equivalent pH on CGA and CA stability and first-pass metabolism of the compounds using a human liver microsomal system containing various phase I and phase II drug metabolizing enzymes, among other ongoing studies.

Among other pharmacodynamic-related studies we are conducting prior to the mouse study is an examination of the intracellular pH modulatory impact of the compounds in both cancerous cells (MDA-MB-231 and MCF7 cells) and normal breast cells (MCF10A cells) and the role of the phytochemicals in the expression and plasma membrane incorporation of some important transport proteins implicated in regulating cancer metabolism. Our in vivo study will involve the use of xenograft mouse model breast cancer cells in examining the anticancer ability of the CGA and CA mixture using the data we have collected not only from in vitro studies, but also from this in silico examination. This study underscores the use of in silico modeling to inform design of in vivo drug efficacy studies more effectively.

## Figures and Tables

**Table 1:** AKT and molecular docking profile.

	Ligand	Akt Binding	Parameters
1	Chlorogenic acid	XYZ coordinates Binding energy Interacting amino acids	25.140333; 6.181083; 8.167583 −8.2 kcal/mol Asp 274; Lys 276; Asn 279; Leu 295; Phe 309; Cys 310; Gly 311
2	Cinnamaldehyde	XYZ coordinates Binding energy Interacting amino acids	27.003800; 6.254100; 9.233900 −5.4 kcal/mol Leu 156; Gly 157; Val 164; Ala 177; Ala 230; Met 281
3	Spirocyclic sulfonamides inhibitor (co-crystallized ligand)	XYZ coordinates	28.185902; 2.786537; 11.33597
4	Capivasertib (the first FDA-approved Akt inhibitor for treatment of breast cancer) The binding energies of FDA-approved drugs ranged from −5.6 to −6.9 kcal/mol [22]	XYZ coordinates Binding energy	30.194838; 2.264703; 12.38245 −8.5 kcal/mol

**Table 2:** PI3K and molecular docking profile.

	Ligand	PI3K Binding	Parameters
1	Chlorogenic acid	XYZ coordinates Binding energy Interacting amino acids	58.764226; −8.98519; 81.31416 −8.5 kcal/mol Ser7; Ile121; Lys672; Ser673; Met 675; His676; Ile713; Asn803; Val845
2	Cinnamaldehyde	XYZ coordinates Binding energy Interacting amino acids	57.478300; −7.86420; 83.815100 −6.2 kcal/mol Trp669; Lys672; Glu674; His676; Val706; Met709; Ile 713; Ile841; Gly842; Asp 843
3	Pyridopyrimidinones inhibitors (co-crystallized ligand)	XYZ coordinates	54.042978; 4.928485; 87.43672

**Table 3:** PDK1 and molecular docking profile.

	Ligand	PDK1 Binding	Parameters
1	Chlorogenic acid	XYZ coordinates Binding energy Interacting amino acids	37.643194; −27.85712; −11.5306 −8.2 kcal/mol Leu88; Val96; Ala109; Lys111; Ser160; Ala 162; Glu209; Leu212; Thr222; Asp223
2	Cinnamaldehyde	XYZ coordinates Binding energy Interacting amino acids	34.57870; −28.64820; −12.19540 −5.8 kcal/mol Leu88; Val96; Ala109; Lys111; Leu159; Ser160; Ala162; Leu212; Thr222
3	7-azaindoles inhibitor (co-crystallized ligand)	XYZ coordinates	34.78813; −27.57596; −12.39844

**Table 4:** Absorption parameters of chlorogenic acid and cinnamaldehyde.

S/N	Parameters	Predicted	Value	Recommendation	Implication
		CGA	CA		
1	Number of rotatable bonds	5	2	≤10, per Veber’s rule	Contributes to molecule conformational flexibility to permeate cell membrane and interact with receptors
2	Molecular weight (g/mol)	354.31	132.16	≤500 g/mol	Small molecules cross cell membrane and have better oral bioavailability
3	Caco-2 permeability (LogP_app_)	−6.49	−4.767	≥0.9	Low intestinal wall permeability
4	P-glycoprotein pump substrate	No	No		Not removed by the efflux pump in the GIT
5	BBB permeability	No	Yes		
6	Octanol/water partition coefficient (Lipophilicity, Log P_o/w_)	−0.39	1.97	0–5	Lower value is suggestive of hydrophilicity or lipophobicity of the compound, which therefore becomes membrane impermeable

**Table 5:** Chlorogenic acid and cinnamaldehyde distribution in systemic circulation.

S/N	Parameters	Prediction		Reference	Implication
		CGA	CA		
1	PPB (%)	64.8	94.9	<90	High plasma protein binding suggests drugs remain unavailable to tissue to elicit therapeutic effects
2	Volume distribution(l/kg)	0.976	0.384	0.4–20	Low volume reaching extravascular tissue based on blood tissue perfusion

**Table 6:** Elimination profiles of chlorogenic acid and cinnamaldehyde.

S/N	Parameters	Prediction		Reference	Implication
		CGA	CA		
1	CL_plasma_ (mL/min/kg)	11.086	3.34	5–15 mL/min/kg Moderate clearance plasma rate	Varied bioavailability in the systemic circulation and plasma removal rate; this may impact volume distribution and half-life
2	T_1/2_ (hr)	1.395	2.758	1–4 h short half-life	Its effect wears off quickly, and 50% is removed quickly from the body.

**Table 7:** Toxicological profiles of chlorogenic acid and cinnamaldehyde.

S/N	Parameters	Prediction	
		CGA	CA
1	Lethal Dose 50 (LD_50_)	5000 mg/kg	1850 mg/kg
2	Hepatotoxicity	Inactive	Inactive
3	Neurotoxicity	Inactive	Active
4	Cardiotoxicity	Inactive	Active
5	Cytotoxicity	Inactive	Inactive
6	Mutagenicity	Inactive	Active
7	Nephrotoxicity	Fairly active	Inactive
8	Pulmonary	Fairly active	Inactive
9	Immunotoxicity	Active	Active

**Table 8:** Drug-likeness of chlorogenic acid and cinnamaldehyde.

S/N	Parameters	Prediction		Optimal Value	Implication
		CGA	CA		
1	Number of H-bond acceptors	9	1	≤10	High number increases polarity and H-bonding with targets but impacts membrane penetration due to hydrophilicity conferment for CGA, but insufficient H-bond forming group in CA, making CA more lipophilic and membrane-penetrating. Undesired H-bonding in CGA, with groups such as P-gp, caused by H-bond donors.
2	Number of H-bond donors	6	0	≤5	
3	Topological polar surface area (TPSA)(Å^2^)	164.75	17.07	0–140 Å^2^	The more surface area covered by polar atoms in a drug, the poorer the drug cell permeability and effectiveness to reach target tissues. 60–80 Å^2^ required to penetrate mammary tissues.
4	Number of rotatable sigma bonds	5	2	≤10	Moderate amount confers appropriate degree of flexibility to the drug, which is important for drug conformational change to cross cell membrane and interact with target at its active site.
5	Number of heavy atoms	25	10	<36	Appropriate number indicative of good binding affinity (target selectivity) and ligand efficiency (ligand binding energy)
6	Number of aromatic heavy atoms	6	6	2–4	Higher increases drug hydrophobicity, which may enhance the ligand binding affinity but may reduce target selectivity via pi orbital stacking with aromatic amino acid side chains of undesired proteins

## Data Availability

Data are available upon reasonable request to the corresponding author.
